# Changes in Transcript Related to Osmosis and Intracellular Ion Homeostasis in *Paulownia tomentosa* under Salt Stress

**DOI:** 10.3389/fpls.2016.00384

**Published:** 2016-03-30

**Authors:** Guoqiang Fan, Limin Wang, Minjie Deng, Zhenli Zhao, Yanpeng Dong, Xiaoshen Zhang, Yongsheng Li

**Affiliations:** ^1^Department of Molecular Biology, Institute of Paulownia, Henan Agricultural UniversityZhengzhou, China; ^2^Division of Plant Biotechnology, Zhengzhou Agriculture and Forestry Scientific Research InstituteZhengzhou, Henan, China

**Keywords:** autotetraploid *Paulownia tomentosa*, osmotic homeostasis, ion homeostasis, salt stress, transcriptome sequencing

## Abstract

*Paulownia tomentosa* is an important economic and greening tree species that is cultivated widely, including salt environment. Our previous studies indicated its autotetraploid induced by colchicine showed better stress tolerance, but the underlying molecular mechanism related to ploidy and salt stress is still unclear. To investigate this issue, physiological measurements and transcriptome profiling of diploid and autotetraploid plants untreated and treated with NaCl were performed. Through the comparisons among four accessions, for one thing, we found different physiological changes between diploid and autotetraploid *P. tomentosa*; for another, and we detected many differentially expressed unigenes involved in salt stress response. These differentially expressed unigenes were assigned to several metabolic pathways, including “plant hormone signal transduction,” “RNA transporter,” “protein processing in endoplasmic reticulum,” and “plant-pathogen interaction,” which constructed the complex regulatory network to maintain osmotic and intracellular ion homeostasis. Quantitative real-time polymerase chain reaction was used to confirm the expression patterns of 20 unigenes. The results establish the foundation for the genetic basis of salt tolerance in *P. tomentosa*, which in turn accelerates Paulownia breeding and expands available arable land.

## Introduction

Greening trees cover much of the terrestrial landscape (He et al., 2011) and are constantly exposed to a wide range of abiotic stresses. According to incomplete statistics ~7% of the world's land including green land is susceptible to salinity (Schroeder et al., 2013), therefore the genetic improvement of trees that can be cultivated in salinity soil become the strong demand (Vinocur and Altman, 2005; Ngara et al., 2012). Current research in this area focus on clarifying the salt adaptation mechanism that will be further contribution to developing salt-tolerant varieties of greening trees.

*P. tomentosa* is an important fast-growing and multi-use tree that is native to China (Bayliss et al., 2005). *P. tomentosa* is able to acclimatize to various soil conditions and climates (Zhu et al., 1986; Dong et al., 2014b; Fan et al., 2015). An autotetraploid of diploid *P. tomentosa* has been induced successfully using colchicines (Fan et al., 2007, 2015). Compared with diploid progenitor, the timber quality and stress tolerance of autotetraploid *P. tomentosa* were better using transcriptome sequencing (Fan et al., 2015). In previous studies, we had shown that autotetraploid Paulownia plants possessed higher drought tolerance than the corresponding diploid plants (Dong et al., 2014c; Xu et al., 2014). The up-regulation of genes involved in the synthesis of abscisic acid (ABA), osmolyte production, transcription factors, and antioxidant were drought tolerance response at transcriptome levels (Dong et al., 2014a,b,c; Xu et al., 2014). However, the salt tolerance mechanism of *P. tomentosa* is still unclear.

According to existing researches, the adverse effects of salinity have been reported to be related to osmotic stress and ion toxicity (Ueda et al., 2006; Ngara et al., 2012; Trivedi et al., 2013; Ziemann et al., 2013). Adaptation mechanisms involving complex gene regulatory networks have evolved to counteract the negative effects of salinity and other stresses during the lengthy evolution of plant species (Munns and Tester, 2008), which mainly include morphological changes, physiological modifications like altered water transport, and metabolite changes like osmolyte, antioxidant enzyme, and hormone synthesis, and molecular regulation like signal transduction (Comstock, 2002; Deinlein et al., 2014; Liu et al., 2015; Wang and Wang, 2015). Some genes such as *HKT1*, a member of the sodium transporter gene families in *Populus euphratica* (Ma et al., 2013), and *NCL*, a nucleolin-encoding gene, in *Arabidopsis thaliana* (Wang et al., 2012) have been reported to be involved in the maintenance of ionic homeostasis under salt stress. Furthermore, transporters are intimately connected with the function and structural integrity of cells and the concentrations of intracellular ions (Cao et al., 2001). Membrane transporters, such as zinc transporters (ZIP) and cation transport regulator proteins, have been found to play important roles in mediating signal transduction, material transport, cell ionic equilibrium and permeability stress (Mentewab and Stewart, 2005; Silva et al., 2007; Wang et al., 2010; Ying et al., 2012; Chalbi et al., 2015). Other genes like basic leucine-zipper transcription factors(bZIP TFs) (Wang et al., 2003; Ying et al., 2012), ras-related nuclear ran GTPase-activating protein (RanGAP) (Ngara et al., 2012) and heat shock proteins (Song and Ahn, 2011; Ma et al., 2013) have been reported to be related to the salt response in plant. However, the genetic basis underlying the adaptation responses associated with maintaining intracellular ion and osmotic homeostasis has not yet been elucidated in detail.

To our knowledge the molecular mechanisms associated with salt adaptation have not yet been reported in Paulownia species so far. In order to broaden this knowledge, we measured the physiological parameters after salt stress. To understand the underlying molecular mechanism, we performed transcriptome sequencing to identify differentially expressed unigenes (DEUs). Quantitative Real-Time PCR (qRT-PCR) was performed to confirm the expression patterns of 20 DEUs. Under the bioinformatics analyses, several pathways, “plant hormone signal transduction,” “RNA transporter,” “protein processing in endoplasmic reticulum,” and “plant-pathogen interaction,” constructed the complex regulatory network to maintain osmotic and intracellular ion homeostasis. Our data highlight the genetic bases of salt tolerance in the *P. tomentosa*, which in turn accelerates Paulownia breeding and expands available arable land.

## Materials and methods

### Plant materials and stress treatments

All plant materials used in this study were obtained from the Institute of Paulownia, Henan Agricultural University, Zhengzhou, Henan Province, China. The tissue culture seedlings of diploid (PT2) and autotetraploid *P. tomentosa* (PT4) had been grown for 30 days in a 16/8 h light/dark cycle at 25 ± 2°C and 130 μmol·m^−2^·s^−1^ illumination intensity. They were transplanted into nutritive bowl (20 cm in diameter at the bottom and 20 cm deep) containing normal garden soil (containing 19 mM NaCl) for 30 days. The plants with uniform growth were selected to transfer individually into nutrition pots (30 cm in diameter at the bottom and 30 cm deep) with trays underneath. The nutrition pots also contained normal garden soil. After 50 days, Paulownia seedlings with consistent growth were selected to be subjected to salt treatment basing on the method of Deng et al. (2013).

The above materials of autotetraploid and diploid *P. tomentosa* were treated with 70 mM NaCl for 0, 5, 10, 15, and 20 days to do phenotype investigation. The morphological changes of *P. tomentosa* leaves at each stress time were observed. The materials of physiological measurements were treated with 0, 35, 70, 105 mM NaCl for 15 days. After 15 days salt stress, mature, fully expanded leaves (the second pair of leaves from the apex shoot) of the salt-treated autotetraploid and diploid *P. tomentosa* were used to conduct physiological measurements, respectively. Meanwhile, we collected the second pair of leaves from the apex shoot of PT2, PT4, and in the same way collected the leaves of PT2 and PT4 that were treated with 70 mM NaCl for 15 days, which were used for the following transcriptome sequencing. We defined the autotetraploid and diploid *P. tomentosa* treated with 70 mM NaCl for 15 days as PT4S and PT2S, respectively. Then the selected leaves of four samples were blended separately. The experiment design processed three repetitions in every step. The four samples were frozen in liquid nitrogen and stored at −80°C for total RNAs isolation and sequencing. Autotetraploid *P. tomentosa* enjoyed the same tissue culture with diploid *P. tomentosa* that warrant the biological replicates (Dong et al., 2014c).

### Phenotype investigation and physiological measurements

Physiological measurements were conducted from 8.30 to 11.00 a.m. Three replicates of each group were defined at the same condition. The percent relative water content (RWC) of leaves were calculated basing on the method described previously (Chen et al., 2010). Seven other physiological indexes (chlorophyll content, malondialdehyde concentration (MDA), relative electrical conductivity (REC), superoxide dismutase (SOD) activity, total soluble protein content, soluble sugar content, and proline content) were determined following the method of Li et al. (Li, 2000). The MSTAT-C computer program and SPSS (v.19.0) were used to analysis the statistic data. The least significance difference test at the 5% level was used to calibrate the mean values of every measured statistics according to Snedecor and Cochran (1967).

### Transcriptome sequencing and assembly

Based on the results of physiological experiment, leaves of the plants (PT2, PT4, PT4S, and PT2S) were selected for the sequencing. A Plant RNA Isolation Kit (AutoLab, Beijing, China) was used to extract the total RNAs; and an RNeasy MiniElute Cleanup Kit (Qiagen, Valencia, CA, USA) was used to concentrate the extracted RNAs. The mRNA was isolated from the total RNAs using oligo(dT)-attached magnetic beads. Fragmentation buffer (Life Technologies, Beijing, China) was added to break the mRNA into short fragments that were used as templates to synthesize the cDNA.

A TruSeqTM RNA Sample Preparation Kit (Illumina, San Diego, CA, USA) was used to construct four (PT2, PT2S, PT4, and PT4S) paired-end libraries. The libraries were sequenced on an Illumina GAII (Illumina) platform. Raw reads were generated, and the paired-end read length is 91nt. The data used in this publication have been deposited in the NIH Short Read Archive database (http://www.ncbi.nlm.nih.gov/sra) and are accessible through SRA accession number SRP058902 (Alias:PRJNA285426). Clean reads were obtained by stringently filtering the raw reads. The filterable reads contain more than 10% of bases with quality scores (*Q*) < 25, non-coding RNAs (such as rRNAs, tRNA, and miRNAs), ambiguous sequences represented as “N,” and adaptor contaminants.

The remaining clean reads were conducted de novo assembly using the Trinity software (Grabherr et al., 2011) (the options: -seqType fq -min_contig_length 200 –group_pairs_distance 250 -min_kmer_cov 2) (http://trinityrnaseq.sourceforge.net/). The assembled fragments of trinity are called unigenes. Unigenes from each sample's assembly can be taken further process of sequence splicing, which was removed redundancy using sequence clustering software to acquire non-redundant unigenes as long as possible. Finally, the non-redundant unigenes were called all-unigenes. The sequences of all-unigenes have been deposited at DDBJ/EMBL/GenBank under the accession GEFV0000000 0. TransDecoder in the Trinity package (the options: -m 100 -G universal -C complete CDSs only -T500) was used to predict the coding region sequences (CDSs) of the assembled transcripts and their corresponding protein sequences.

The generated clean reads of duplicate controlled diploid and autotetraploid *P. tomentosa* have been deposited in the NIH Short Read Archive database (http://www.ncbi.nlm.nih.gov/sra) and are accessible through SRA accession number SRP031515, including PT2 (SRX 365528, SRR1013710), PT4 (SRX365650, SRR10137120). The FPKM methods (Mortazavi et al., 2008) were used to calculate the expression of the unigenes. The correlation coefficients of logarithmic values of the expression of both the duplicated samples were then calculated. If the absolute value of pearson correlation coefficient (|*r*|) ≥0.8, strongly linear correlation between the probe set pairs exist; if |*r*| ≥0.5 and < 0.8, moderate linear correlation between the probe set pairs exist; if |*r*| ≥0.3 and < 0.5, low linear correlation between the probe set pairs exist; if |*r*| < 0.3, no linear correlation between the probe set pairs exist (Häne et al., 1993; Wang et al., 2015).

### Functional annotation and analysis of unigenes

All the unigenes were used as queries in local BLASTX searches (*E*-value threshold of 1.0 × 10^−5^) against NCBI's non-redundant protein database (Nr) (http://www.ncbi.nlm.nih.gov/), Swiss-Prot (http://www.ebi.ac.uk/swissprot/), Eukaryotic Orthologous Groups (KOG), and Gene Ontology (GO). Proteins with highest ranks in blast results were taken to decide the coding region sequences (CDSs) of unigenes. Based on the annotations of the known sequences in the BLAST alignments, the Blast2GO tool (Conesa et al., 2005) was used to assign GO terms to the assembled unigenes; and the WEGO tool (Ye et al., 2006) was used to classify these GO terms. The unigenes were also searched against the Kyoto Encyclopedia of Genes and Genomes (KEGG) database using BLASTp.

### Gene expression quantification and differential expression analysis

Quantification of unigene expression in the four libraries were characterized using RNASeq by Expectation Maximization (RSEM) (http://deweylab.biostat.wisc.edu/rsem/) (Li and Dewey, 2011) to obtain the read counts for each unigenes in each of the four libraries. Fragments per kilobase per million reads (FPKM)(Mortazavi et al., 2008) values were used to compare the expression levels of the unigenes between two libraries as well as a measure of relative expression levels in a single library, which were converted from the read counts obtaining homogenization by Trimmed Mean of *M*-values (Robinson and Oshlack, 2010). The Bio-conductor edgeR (http://www.bioconductor.org/packages/release/bioc/html/edgeR.html) (Robinson et al., 2010) was used to compute the differentially expressed value of the unigenes via the internal normalization step based on the read counts that were calculated directly from the transcriptome data by RSEM. Among all the unigenes, those with thresholds of an absolute log2Ratio value≥1, *p* ≤ 0.05, and false discovery rate (FDR) ≤ 0.001 were defined as DEUs using EdgeR software. Finally, the DEUs from four comparisons (PT2S vs. PT2, PT4S vs. PT4, PT4 vs. PT2, and PT4S vs. PT2S) were identified. Additionally, the common DEUs between PT2S vs. PT2 and PT4S vs. PT4, the common DEUs between PT4S vs. PT2S and PT4 vs. PT2, the common DEUs among four comparisons were also identified with the above methods. The DEUs were selected to conduct the GO and KEGG enrichment analysis which were performed using hypergeometric tests. The Bonferroni correction was used to adjust the *p*-value. DEUs with *p* < 0.05 were considered as significant enriched in GO terms and KEGG pathways.

### qRT-PCR verification of unigenes related to salt response

The materials of qRT-PCR were PT4 and PT2 which were treated 0, 5, 10, 15, and 20 days with 70 mM NaCl. The total RNAs were extracted from the leaves of the control and treatment groups referring to the above mention. First-strand cDNA was reverse transcribed using an iScript cDNA Synthesis Kit (Bio-Rad, Hercules, CA) according to the manufacturer's instructions. Twenty DEUs that were predicted to be related to salt adaptation were selected for qRT-PCR analysis. Primers (Table 1) were designed using Beacon Designer version 7.7 (Premier Biosoft International, Ltd., Palo Alto, CA, USA). The qRT-PCR reactions were run in SsoFast EvaGreen Supermix (Bio-Rad) with 1 μL cDNA template in a standard 20-μL reaction. The following PCR cycles were used: 2 min at 95°C, followed by 40 cycles of amplification for 15 s at 95°C, then annealing for 15 s at 57°C. Three replicates were analyzed for each DEU. These reactions were run using a CFX96™ Real-Time PCR Detection System (Bio-Rad). 18S rRNA was regarded as the internal control. The 2^−ΔΔCt^ method was used to calculate the relative expression levels.

**Table 1:** **Primers of quantitative RT-PCR analysis of 20 selected differently expressed unigenes**.

**GeneID**	**Sense Primer**	**Anti-sense Primer**
m.8589	CCACTTCATTCTTCCTTCCAC	CAACGCCCTCTACACTCG
m.8961	CCCATCACAATCCCAACCATC	GAGCAGCCAAGAGCAATAGG
m.17708	AAGAGGCTGTTGATGATG	GTGAGACTCCAATTATTACC
m.13434	CAATCATCCAGCCATTCAAG	AGTGTAACCTCCTAATGTAGC
m.2890	AGGATGGAGTGCTGAGGATTAC	CGGAGGTGGTGGCTGTTC
m.23439	ACCACCATTACCACCACCTC	TGTTCTTTCCCTTAGTTTCCATCC
m.34904	CAAGGTTAGGCATTCACTCTG	CCGCACATCATCAAGTATTCC
m.35058	GGACCGAAGAAGGATTACAC	ATCACCACAGAATAGCATACC
m.20494	GCTGACTACTACGCACATCC	GCCACCTACATCACAAGTTATATC
m.42732	AGCAGTCTCAATAGCAATGG	TGTGTCAGGTCCTCTTCC
m.2899	TCTCCACTATCCACACTACTACC	GCTTAACATCCTCCTCTTTCCC
m.20666	TACAAAGGTCGTGGTCTCC	TGGCAGCATTATCAATCTCC
m.14413	GGCGATGCTGAGAGACTGTTG	GCTTCCTCCTCCGACCTTCC
m.6898	TGACAACACGCCAAGCAG	CAAGGACAACGAGCAGACC
m.45786	CTTGCTCCGTCACCACCATTAG	TCTGCTTCATCGTCGCCAATATC
m.34763	GCCAATGAAGTAACAGAAG	CAATGAGAGCACAATAAGC
m.17951	CAACCTCAGCAGCCTCAG	AAGCATTCGTCATTCCAACC
m.49968	GGCACATCACAGACACAG	ACACCACCTTCCTCATCC
m.5670	CCTGAACATTCGGAACCTCTG	GAACAATCTCACGGCATTATACG
m.1327	CGAAGTGGTGGGATTTGTTTG	GCTATGGTGCCTCTTGGATG

## Results

### Morphologic changes and physiological responses of autotetraploid and diploid *P. tomentosa* to salt stress

The phenotype responses of PT2 and PT4 were investigated under 70 mM NaCl treatment. After 5 days salt treatment, the leaves showed no significant phenotype changes. However, after 10 days salt treatment, the leaves of PT4 and PT2 displayed slight wilt; overall the leaves of PT4 were more turgid than those of PT2. After 15 days salt treatment, the leaves of PT4 were less wilted than those of PT2, which showed stunted growth. After 20 days salt treatment, the PT2 and PT4 plants were both heavily wilted (Figure S1). The above phenomena might be result from different ploidy. According to this result, we determined the salt stress time of *P. tomentosa* that was used to sequencing.

After SPSS analysis of all the physiological indicator values, the RWC and chlorophyll content declined in both accessions as the salinity increased. The decline was possibly induced by weakened water absorption ability under salt environment and led to the wilting of leaves. The REC and MDA content increased as a result of lipid peroxidation mediated by ROS that were generated with the salt concentration increase in both PT2 and PT4; however, the mean levels were lower in the leaves of PT4 compared with PT2 over the entire treatment period. The SOD activity and soluble protein content of PT4 and PT2 leaves both showed increase when the treatment ranged from the control sample to 70 mM NaCl sample, decreased at 105 mM NaCl site; while SOD activity was higher in PT4 than in PT2 at 70 mM NaCl site. SOD acted as a primary ROS scavenger which might be related to the above lower values of REC and MDA. The proline, soluble protein, and soluble sugar contents in the leaves of PT4 and PT2 tended to increase as the salt concentration increased; but their levels were higher in PT4 than in PT2, which might help maintain the osmotic equilibrium of the cells (Figure S2).

### Sequencing and assembly

**Figure 1:**
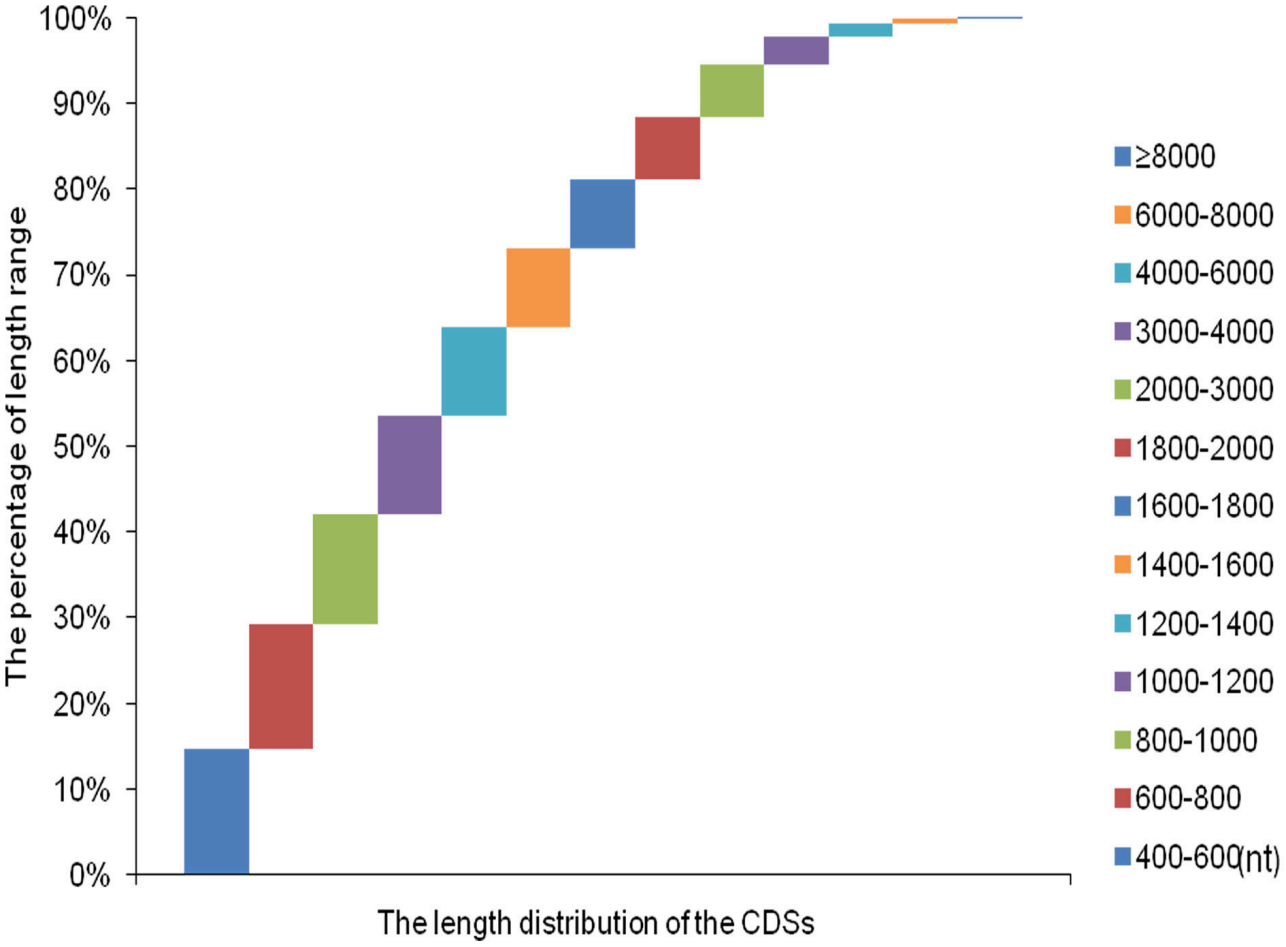
**The length distribution of the coding sequence**.

Over 273 million raw reads with a mean length of 184 nt from the four libraries (PT2, PT4, PT2S, PT4S) were generated by Illumina sequencing. After filtering out low quality reads, more than 256 million clean reads with a mean Q20 percentage of 85.32% and GC content of 44% were obtained. The sequencing and assembly results were summarized in Table 2. Then 256,438,786 clean reads from four accessions were assembled into 167,145 all-unigenes (these unigenes with 205,180,270 nt, a mean length of 2455 nt, and N50 of 4426 nt). The length distribution of unigenes ranged from 400 to 10000 nt (Figure S3) and 15,967 unigenes was longer than 6000 nt. Predictions of protein coding regions revealed 350 CDSs that were longer than 8000 nt. The length distribution of the CDS was shown in Figure 1. These results demonstrated the high quality of the assembly. In addition, the correlation coefficient of the expression of diploid and autotetraploid *P. tomentosa* has been investigated in our previous researches (Dong et al., 2014a). The data used in this correlation coefficient analysis have been deposited in the NIH Short Read Archive database (http://www.ncbi.nlm.nih.gov/sra) and are accessible through SRA accession number SRP031515 (Alias:PRJNA222862), including PT2(SRX 365528, SRR1013710), PT4(SRX365650. SRR10137120). Both duplicates showed linear correlations with their corresponding ones. The Pearson *r*-values of the diploids and autotetraploid were 0.81 and 0.87, respectively. The results indicated that there were strongly linear correlations among diploid and autotetraploid *P. tomentosa*.

**Table 2:** **Overview of the sequencing and assembly of the transcriptome of *Paulownia tomentosa***.

**Statistics of data production**	**PT2**	**PT4**	**PT2S**	**PT4S**
**READS**				
Number of raw reads	49,134,426	46,924,464	84,134,270	93,422,042
Number of clean reads	46,031,492	44,265,604	77,819,320	88,322,370
Total nucleotides (nt)	8,808,974,514	8,593,233,246	13,451,321,008	15,613,216,698
Q20 percentage (%)	89	91	79	83
GC percentage (%)	44	44	44	44
Average length (nt)	191	194	173	177
**ALL UNIGENES**				
Number of all unigenes		167,145		
Total nucleotides (nt) in all unigenes		205,180,270		
Length of N50 (nt)		2455		
Average length of all unigenes (nt)		4426		

### Functional annotation

The 167,145 assembled unigenes were aligned against the sequences in four public databases (Nr, Swiss-Prot, KOG, and GO). A total of 33,778 (20.21%) and 26,139(15.64%) unigenes had homologous sequences in the Nr and Swiss-Prot databases, respectively. A total of 12,790 (7.65%) unigenes were categorized into 25 specific functional groups in the KOG database (Figure S4). A total of 15,385(9.20%) unigenes were assigned to 38 functional categories in the GO database under the three main functional categories (biological process, cellular component, molecular function) (Figure S5). A total of 8663(5.18%) unigenes were mapped to 307 reference pathways (Table S1) that contain metabolic pathways potentially involved in salt response. Many unigenes were annotated in “carbohydrate metabolism,” “signal transduction,” and “genetic information processing pathways”. The abundant information of function annotation in all-unigenes would lay a good foundation for the following analysis of differentially expressed genes.

### Analysis of DEUs related to salt tolerance

**Figure 2:**
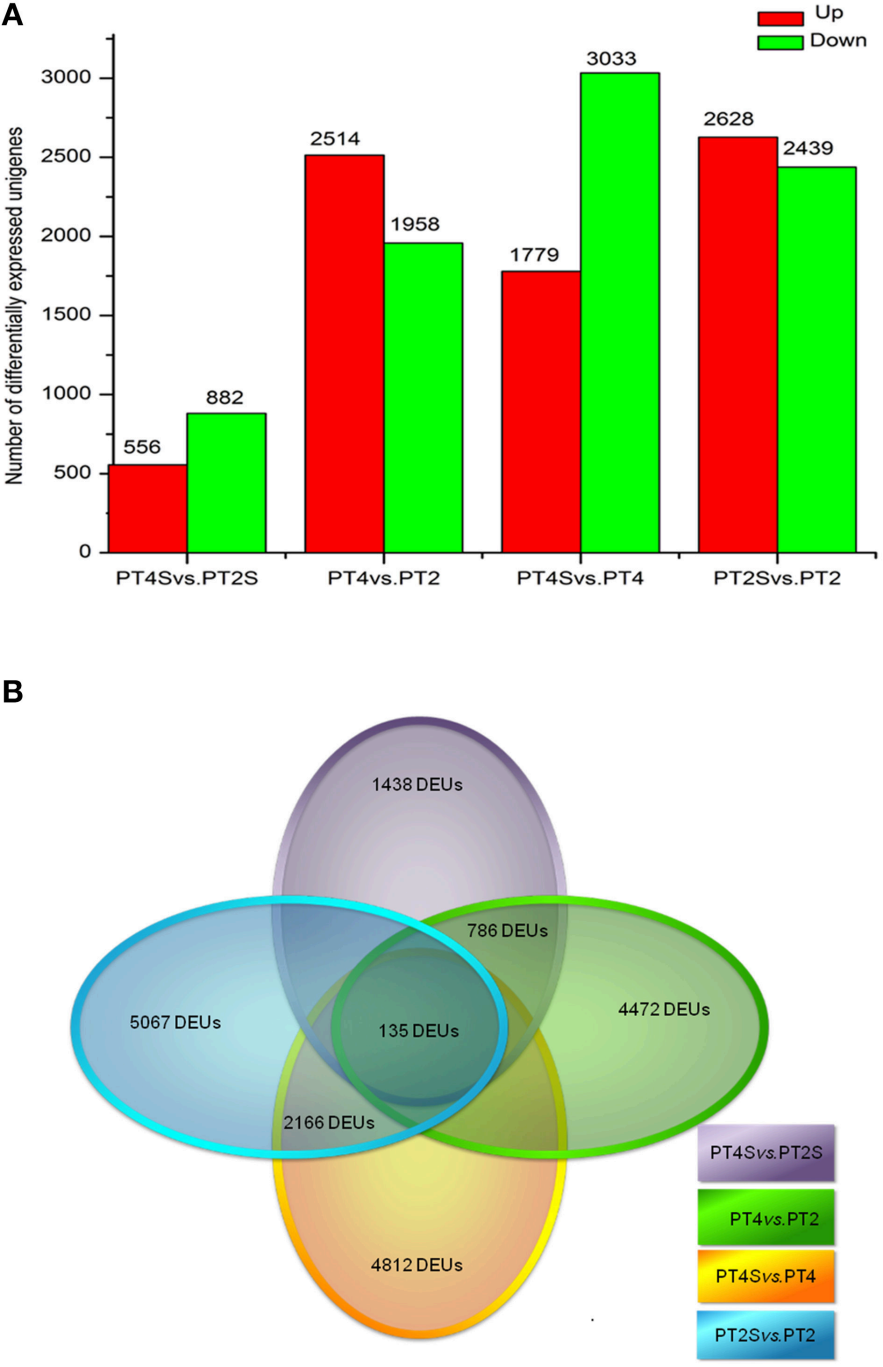
**(A)** The differently expressed unigenes in PT4 vs. PT2, PT4S vs. PT2S PT2S vs. PT4S and PT4S vs. PT4. **(B)** The differential consistently expressed unigenes among four comparisons.

**Figure 3:**
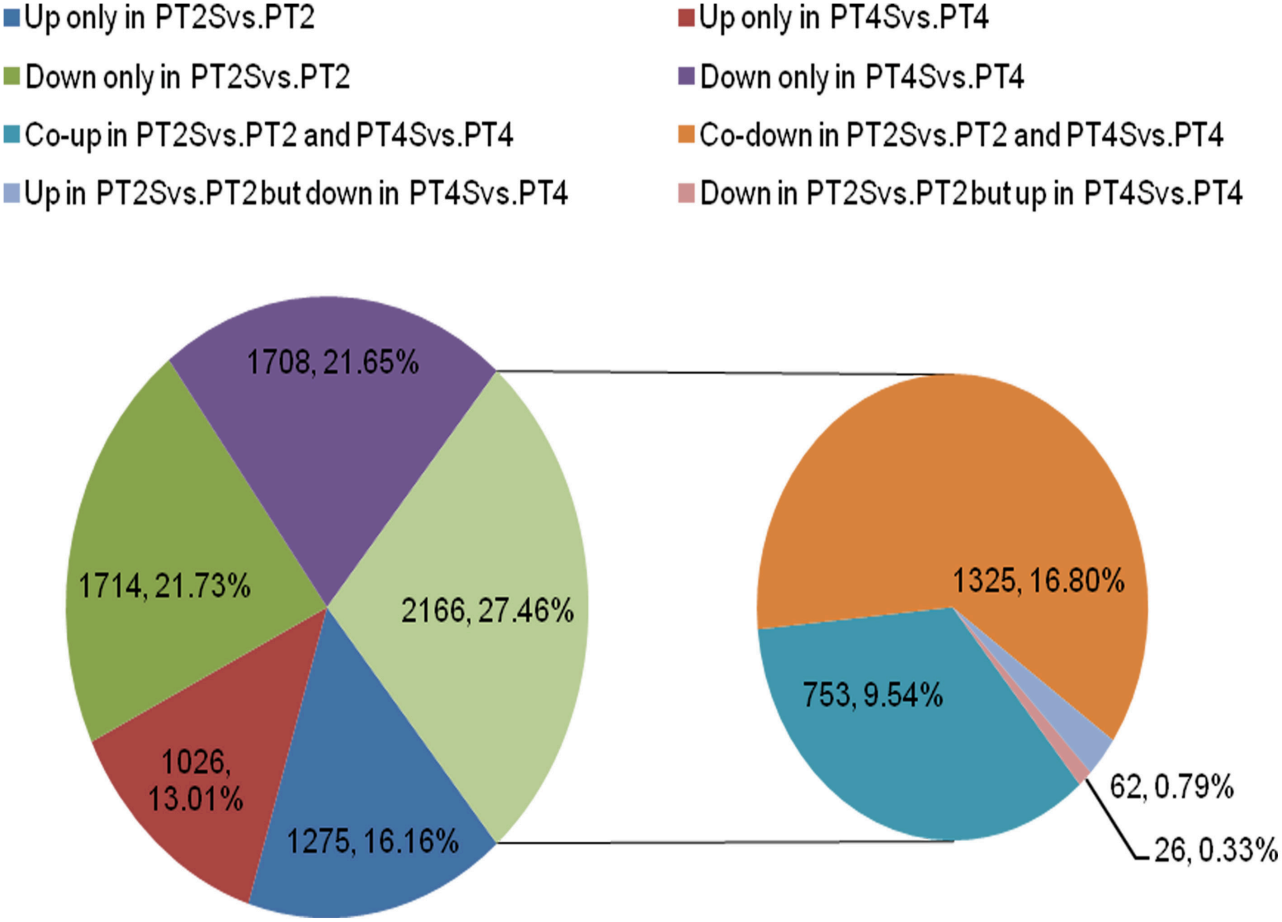
**The differentially expressed unigenes in two comparisons PT2S vs. PT2 and PT4S vs. PT4**. Two thousand one hundred sixty-six differentially expressed unigenes were clustered into eight categories according to their expression patterns.

Among the 167,145 unigenes, 5067 unigenes in PT2S vs. PT2, 4472 unigenes in PT4 vs. PT2, 1438 unigenes in PT4S vs. PT2S and 4812 unigenes in PT4S vs. PT4 were differentially expressed, respectively (Figure 2 and Table S2). In addition, 2166(1.30%) unigenes were consistently differentially expressed in PT2S vs. PT2 and PT4S vs. PT4 (Figure 3 and Table S3A). It was therefore concluded that 7713 DEUs might be salt responsive unigenes, including 2901 DEUs specific to PT2S vs. PT2 and 2646 DEUs specific to PT4S vs. PT4. The DEUs coding SOD, peroxidase (POD), S-(hydroxymethyl) glutathione dehydrogenase, and cytochrome P450 were identified, which involved in ROS scavenging (Oka et al., 2015). The DEUs coding aquaporin Aqp2 related to water uptake, ABC transporter, and proline rich protein related to osmotic adjustment were positively answer to salt treatment; Disease resistance response protein 206 (*DRR206*) involving in disease resistance and bZIP TFs related to ABA signal transduction were up-regulated after salt stress; Ran GAP1 were up-regulated consistently in *P. tomentosa* after salt treatment; the chlorophyll a-b binding proteins that enriched into photosynthesis were down-regulated (Table 3). The DEUs coding ZIPs were all up-regulated in PT4S vs. PT4 and all down-regulated in PT2S vs. PT2 indicating that these unigenes were stress-response positively only in autotetraploid *P. tomentosa*. These results indicated that the different ploidy *P. tomentosa* plants all possessed the molecular basis of salt adaptation.

**Table 3:** **The differential consistently expression unigenes in PT2S vs. PT2S and PT4S vs. PT4 involved in salt response**.

**GeneID**	**logFC (PT2S/PT2)**	**Significant (PT2S/PT2)**	**logFC (PT4S/PT4)**	**Significant (PT4S/PT4)**	**NR annotation**
m.919	3.13	Up	2.12	Up	Peroxidase
m.1815	2.97	Up	2.04	Up	S-(hydroxymethyl)glutathione dehydrogenase
m.43938	2.11	Up	1.51	Up	ABC transporter G family member 15
m.43934	2.19	Up	1.67	Up	ABC transporter G family member 15
m.43935	2.18	Up	1.69	Up	ABC transporter G family member 15
m.1327	2.45	Up	2.13	Up	ABC transporter G family member 15
m.5670	4.41	Up	3.57	Up	ABC transporter G family member 31
m.5671	2.93	Up	4.08	Up	ABC transporter G family member 31
m.2899	1.59	Up	1.65	Up	Aqp2 protein,water channel protein
m.1915	3.77	Up	−2.17	Down	Proline rich protein 1
m.8589	1.56	Up	−2.3	Down	SOD2
m.10024	−1.8	Down	3.54	Up	Cytochrome P450 76C4
m.8829	−6	Down	−4.39	Down	Chlorophyll A/B binding protein, putative
m.31296	−4.3	Down	−3.15	Down	Chlorophyll A/B binding protein
m.31295	−4.2	Down	−3.27	Down	Chlorophyll A/B binding protein
m.6912	−1.9	Down	−2.53	Down	Chlorophyll A/B binding protein
m.8831	−5.5	Down	−4.22	Down	Chlorophyll A/B binding protein
m.12428	−1.7	Down	−1.37	Down	Chlorophyll a/b binding protein
m.31294	−2.8	Down	−2.53	Down	Chlorophyll a/b-binding protein
m.31290	−3	Down	−3.26	Down	Chlorophyll a-b binding protein 13, chloroplastic
m.31292	−4.2	Down	−3.18	Down	Chlorophyll a-b binding protein 21, chloroplastic
m.12427	−2	Down	−1.71	Down	Chlorophyll a-b binding protein 8, chloroplastic-like
m.22840	−2	Down	−2.3	Down	Chlorophyll a-b binding protein P4, chloroplastic-like
m.31293	−3.5	Down	−3	Down	Light-harvesting complex II protein Lhcb3
m.31289	−4.2	Down	−3.18	Down	light harvesting chlorophyll a/b-binding protein
m.31288	−4.2	Down	−3.39	Down	Light harvesting chlorophyll a/b-binding protein
m.31291	−4.3	Down	−3.22	Down	Light harvesting chlorophyll a/b-binding protein
m.31297	−4.2	Down	−3.33	Down	Light harvesting chlorophyll a/b-binding protein

Some particularly responsive DEUs were identified. For example, the 60S ribosomal export protein NMD3-like isoform 1(NMD3) were up-regulated only in PT4S vs. PT4 (Table S4). Thus our results indicated that *P. tomentosa* plants had common salt responsive regulation but autotetraploid-specific adjustments were also presenced. From Table S4, bZIP TFs were aligned with protein dimerization activity (GO:0046983) and regulation of transcription, DNA-templated (GO:0006355). NMD3 is annotated into structural constituent of ribosome (GO:0003735) from molecular function, which is related to translation (GO:0005840) in the terms of biological pathway, and as to cellular component which locate in the ribosome (GO:0006412). ZIPs were matched into metal ion transmembrane transporter activity (GO:0046873), membrane (GO:0016020), and transmembrane transport (GO:0055085). ABC transporter were mapped to ATPase activity (GO:0016887) and membrane (GO:0016020). The results suggested that *P. tomentosa* conducted the network of salt response.

After removing the consistent DEUs from the control libraries (PT4 vs. PT2), the treatment libraries (PT4S vs. PT2S) could be detected the salt responsive unigenes in different ploidy *P. tomentosa*. We found 786 (0.47%) unigenes were consistently differentially expressed in PT4 vs. PT2 and PT4S vs. PT2S. when the different ploidy factors were excluded; we identified 652 DEUs that were present only in PT4S vs. PT2S (Table S5 and Figure S6). The DEUs reflected the difference in salt response between the autotetraploid and diploid *P. tomentosa* plant. The up-regulated DEUs in PT4S vs. PT2S encoded xyloglucan endotransglucosylase/hydrolase protein 9, transferring glycosyl groups, hexose transporter-like protein, and amino acid permease were involved in the soluble protein and soluble sugar contents (Table 4). Soluble protein and soluble sugar play important roles in maintaining the intracellular and extracellular osmotic balance. Additionally, 135 DEUs were identified in all four comparisons (PT2S vs. PT2, PT4S vs. PT4, PT4 vs. PT2 and PT4S vs. PT2S; Tables S3B,S3C).

**Table 4:** **The only up-regulated DEUs related to the soluble protein and soluble sugar contents in PT4S vs. PT2S**.

**GeneID**	**logFC (PT4S/PT2S)**	***P*-Value**	**FDR**	**Significant**	**NR**
m.11012	2.23	6.24E-05	0.003010308	Up	Amino acid permease
m.11013	1.94	0.000633354	0.020903579	Up	Amino acid permease
m.47668	1.56	0.001422639	0.038352504	Up	Hexose carrier protein HEX6
m.23131	1.67	0.000872312	0.025844756	Up	Transferase, transferring glycosyl groups
m.24915	2.59	1.51E-06	0.000116365	Up	Xyloglucan endotransglucosylase/hydrolase 9
m.44551	1.53	0.001468968	0.03928733	Up	Probable galactinol–sucrose galactosyltransferase 2
m.3403	2.91	0.0001553	0.006607435	Up	Phosphofructokinase
m.20603	1.85	0.000108737	0.004899587	Up	Beta-glucosidase 12
m.19923	2.04	2.08E-05	0.001180838	Up	UDP-glycosyltransferase 79B6
m.19928	2.20	5.06E-06	0.000350842	Up	UDP-glycosyltransferase 79B6
m.15211	2.02	8.43E-05	0.003921683	Up	UDP-glycosyltransferase 86A1

### GO and KEGG enrichment analyses of DEUs

**Figure 4:**
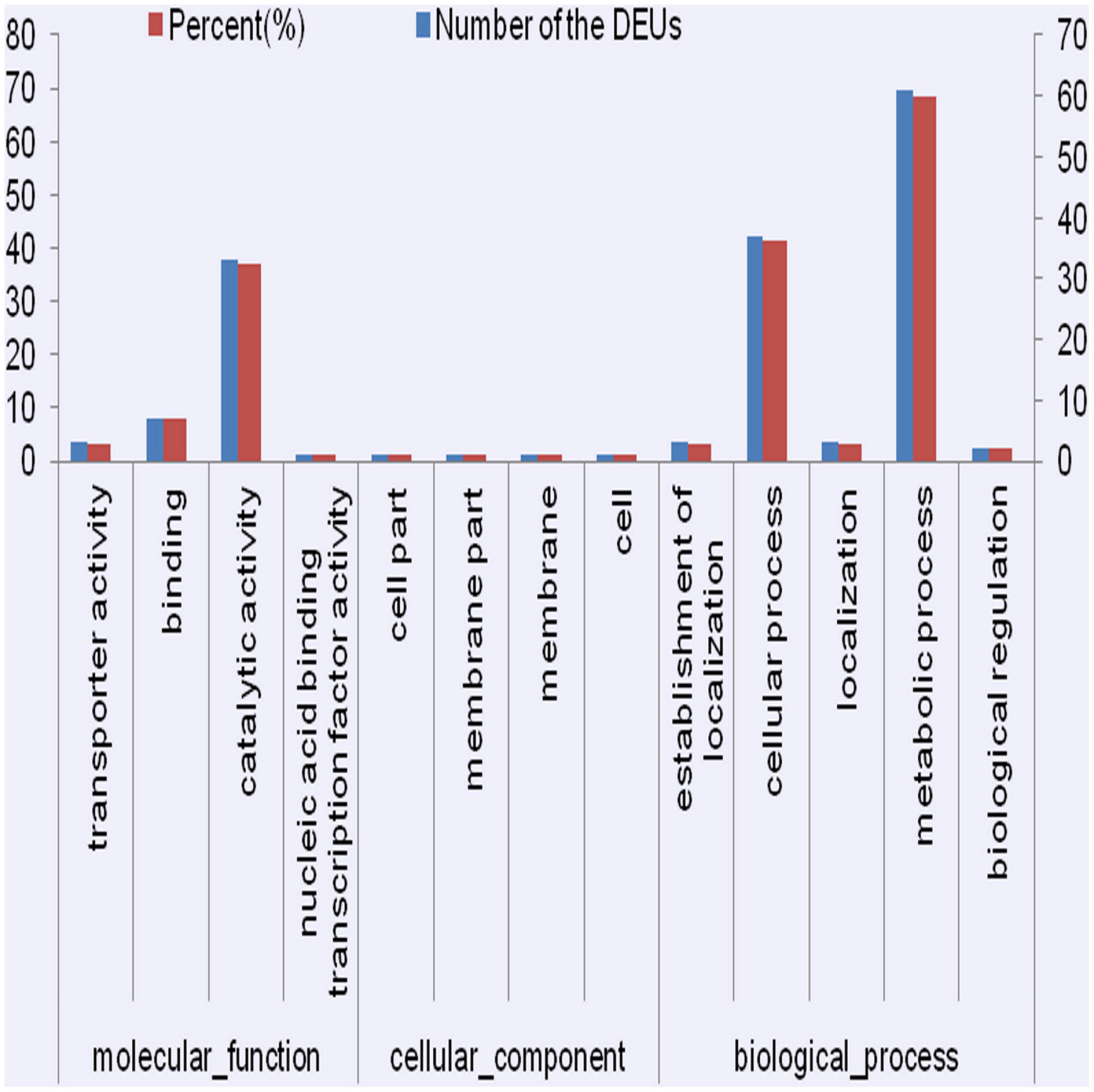
**The consistently differential expression unigenes from four comparisons were enriched in GO terms**.

The GO enrichment analysis allowed us to detect significantly enriched functional categories of the DEUs (Figure 4, Figures S7, S8). Many DEUs enriched into Binding, catalytic activity, cellular process, and metabolic process. After detailed classification of molecular function and biological progress, the enriched GO terms of co-regulated DEUs in PT2S vs. PT2 and PT4S vs. PT4 fell into 92 categories. Many of these DEUs were enriched into transporter activity, catalytic activity and nucleic acid binding transcription under molecular function. In the PT4 vs. PT2 and PT4S vs. PT2S comparisons, the enriched GO terms of co-regulated DEUs were divided into 48 categories. The enriched function terms in these comparisons were similar. Under the biological progress category, many DEUs were enriched consistently in biological regulation and metabolic process, and also some DEUs were enriched in signaling. The enrichment GO terms of 135 co-regulated DEUs in four comparisons were classified into 25 categories (Table S6). Oxidoreductase activity and catalytic activity and carbohydrate biosynthesis were identified.

To gain insight into the underlying metabolic network related to salinity resistance, we carried out KEGG pathway enrichment analysis of the DEUs (*Q* ≤ 0.05). The 1431 DEUs that were co-regulated between PT2S vs. PT2 and PT4S vs. PT4 were enriched into 132 pathways (Table S7A), the 693 common DEUs in PT4 vs. PT2 and PT4S vs. PT2S comparisons were enriched into 76 pathways (Table S7B), and the 194 consistent DEUs in the four comparisons were enriched into 29 pathways (Table S7C), among which the four pathways, namely “Ras signaling pathway” (ko04014), “cAMP signaling pathway”(ko04024), “RNA transport”(ko03013), and “protein processing in endoplasmic reticulum”(ko04141), were identified. The “AMPK signaling pathway” and “neurotrophin signaling pathway” were identified only in PT4S. The concentration in public enrichment pathways showed the overall consistency of the samples under salt treatment.

### Validation of sequencing expression levels by qRT-PCR analysis

**Figure 5:**
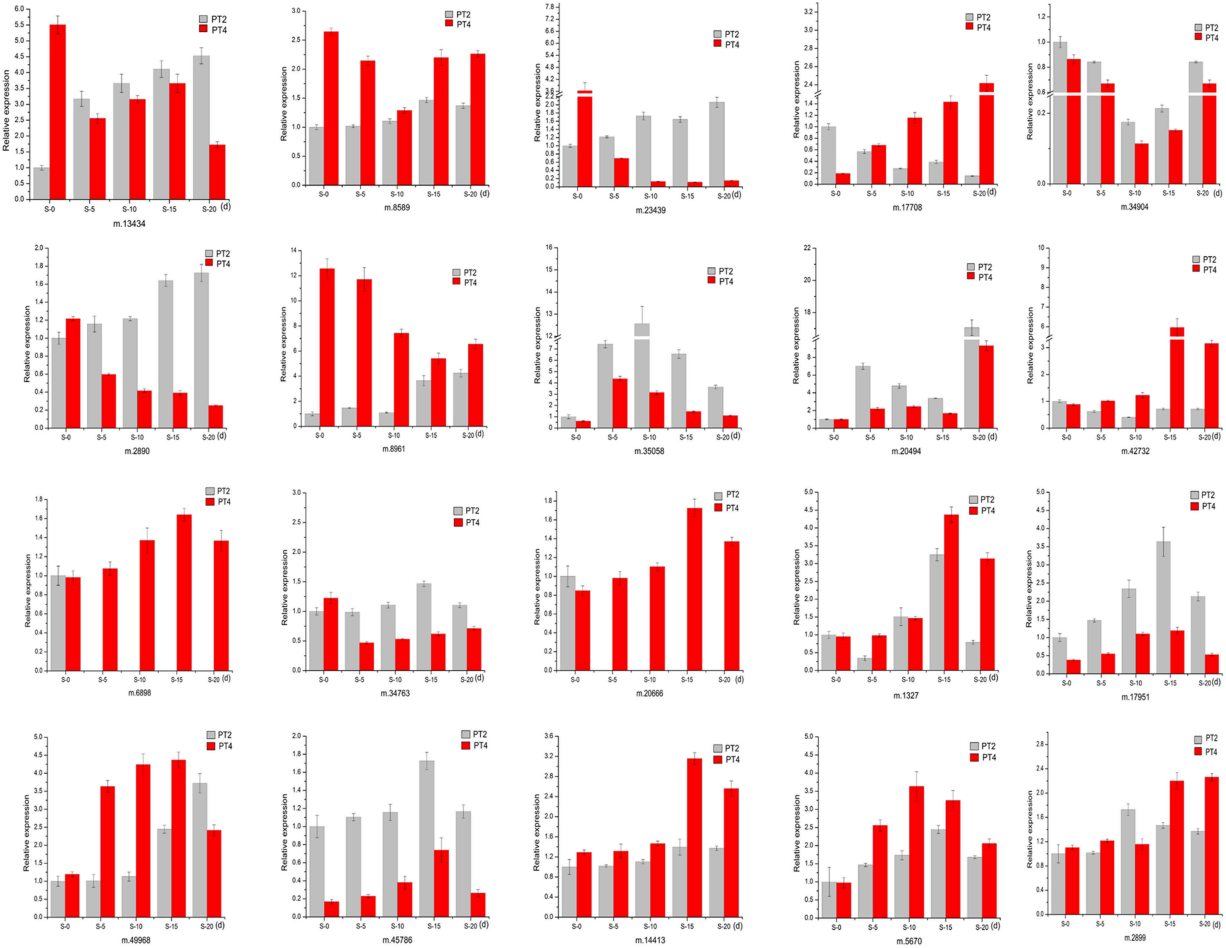
**Quantitative Real-Time PCR (qRT-PCR) analysis of 20 selected differentially expressed unigenes**. 18S rRNA was used as the internal reference gene. For each group, the PT2(S-0) expression level was considered as 1.00, and other samples were normalized accordingly. Standard error of the mean for three technical replicates is represented by the error bars. S-0, 0 day, 70 mM salt-treated for PT2, and PT4; S-5, 5 days, 70 mM salt-treated for PT2 and PT4; S-10, 10 days, 70 mM salt-treated for PT2, and PT4; S-15, 15 days, 70 mM salt-treated for PT2, and PT4; S-20, 20 days, 70 mM salt-treated for PT2, and PT4.

To validate the reliability of our sequencing technology, 20 DEUs from different ploidy and different stress time under 70 mM NaCl (Table 5) were selected for qRT-PCR analysis (Figure 5). The results indicated that 20 genes were consistent between the qRT-PCR and the transcriptome analyses at S-15 sites. In the Table 5, the above 10 DEUs that were randomly selected from Table S3C for qRT-PCR analysis to explore the unigenes' spatio-temporal expression, which confirmed the results of sequencing technology with almost all the same expression tendency; The last 10 DEUs were selected for qRT-PCR analysis because they were important genes related to osmotic and intracellular ion homeostasis. Compared with the control sample (PT2), PT4 had a higher expression of phospholipase D (PLD, m.34763), which was annotated into two pathways of “ras signaling pathway,” “cAMP pathway,” while after salt treatment it showed the low expression level. In addition, the expression levels of PLD in PT2 at four stress sites were all higher than those in PT4. The expression levels of SOD2 (m.8589) in PT4 (S-5, S-10, S-15, and S-20) were higher than those in PT2, which suggested that *P. tomentosa* had a universal salt response, but also varied from different ploidy. ZIPs (m.17708) in PT4 had a lower expression than that in PT2, while it showed the increased expression in PT4 after salt treatment and its expression levels were higher, particularly at S-15 and S-20 sites, than those in PT2. In each salt treatment periods, PT2 showed gradual decrement trend. It was therefore concluded that ZIPs responded positively to salt stress in PT4. The Ran GTP1 (m.14413) manifested the higher expression after salt treatment, and at every stress site, its expression level in PT4 was higher than that in PT2. Among these unigenes, ABCG31 (m.5670), bZIP37 TFs (m.17951), and ZIP4 precursor (m.49968) showed higher expression after salt treatment than the control samples, respectively. The expression of bZIP57 TFs (m.6898) in PT2 were lower than that in PT4; after salt treatment, its expression in PT4 was increased. DRR 206 (m.45786) and Aqp2 protein (m.2899) had high expressed under salt stress in different ploidy *P. tomentosa*, while Aqp2 showed higher expression in PT4 (except S-10) than those in PT2, but not DRR 206 (m.45786). NMD3 (m.20666) was found only up-regulation in PT4 under salt stress. The ABCG15 (m.1327 in PT2) and m.34904 in ever stress sites showed a trend of fall first then rise. The inconsistency trends also contain m.45786, m.5670, m17951 in PT2, and PT4 (rise first then fall), which was probably because transcriptome was more sensitive in the detection of low abundant transcripts and small changes in gene expression than qRT-PCR method. The results indicated the validity of the sequencing method.

**Table 5:** **Annotation of 20 selected differentially expressed unigenes**.

**GeneID**	**Description**
m.8589	SOD2
m.8961	ER glycerol-phosphate acyltransferase
m.17708	Zinc transporter 1
m.13434	Trans-cinnamate 4-monooxygenase
m.2890	22.7 kDa class IV heat shock protein
m.23439	Very-long-chain enoyl-CoA reductase
m.34904	Trehalose 6-phosphate phosphatase
m.35058	GABA transporter 1
m.20494	Aminoadipic semialdehyde synthase
m.42732	Wall-associated receptor kinase-like 9
m.14413	RAN GTPase-activating protein 1 isoform 2
m.34763	Phospholipase D
m.1327	ABC transporter G family member 15
m.2899	Aqp2 protein
m.20666	60S ribosomal export protein NMD3 isoform 1
m.6898	bZIP transcription factor 57
m.45786	Disease resistance response protein 206
m.17951	bZIP transcription factor 37
m.49968	Zinc transporter 4
m.5670	ABC transporter G family member 31

## Discussion

Salinity, one of many abiotic stress factor, restricts plant biomass and increases the risk to sustainable agriculture and forestry worldwide (Ma et al., 2013). Salt treatment caused the changes of *P. tomentosa*'s leaves in morphology and physiological parameters (Figures S1, S2), but these changes cann't cause a fatal harm for its growth (Deng et al., 2013). The underlying molecular base was investigated via transcriptome sequencing that confirmed by qRT-PCR analysis. The transcriptome data yielded 167,145 assembled unigenes, many of which were differentially expressed. In addition, some overlapped DEUs were discovered. The comparison of salt treatment and control accessions of different ploidy *P. tomentosa* dwindle the range of DEUs group, which facilitate identifying genes that are likely related to salt response and adaptation. Some pathways, “Plant hormone signal transduction” (ko04075), “RNA transport” (ko03013), and “protein processing in endoplasmic reticulum” (ko04141), were significantly enriched metabolic pathways, which may help reform the complex regulatory network to maintain osmotic and intracellular ion homeostasis.

### Morphologic and physiological parameters changes modulate osmotic equilibration

In our study, the soluble substances, such as soluble sugar, proline content and soluble protein, were elevated because of salt stress (Figures S1, S2), which maintained the high osmotic pressure in cytoplasm promising the water absorbance and the normal physiological function of cells under salt treatment. The morphological changes, wilting and inward rolling, decrease transpiration by reducing the leaf surface (Wang and Wang, 2015), which possibly mitigate slightly the decreased RWC of leaves in the early stages of salt exposure, but did not prevent wilting in the later salt-stress stage in spite of Aqp2 genes was up-regulated under transcriptome level and higher expression from qRT-PCR analysis after salt stress. Meanwhile, wilting leaves have been reported to increase proline formation (Stewart and Lee, 1974). However, chlorophyll content decreased and stomatal closure in those wilting leaves (Muller and Santarius, 1978; Rao and Rao, 1981; Maslenkova et al., 1993) pose a threat to plant biomass.

### Up-regulated *RanGAP1* and *NMD3* were enriched significantly in the RNA transport pathway

The up-regulated DEU coding RanGAP1 was enriched significantly into “RNA transport pathway” (ko03013). RanGAP1 plays an important role in plant growth and development because it associates with the nuclear pore complex and participates in the regulation of nuclear transport (Haberland, 1999), mitotic spindle assembly, and the cell cycle (Oussenko et al., 2011), and the formation of the envelope (Zhang and Clarke, 2001). It interacts with Ras-related nuclear protein 1 to regulate guanosine triphosphate (GTP)-binding and exchange. The aim RNA combined with RanGTP is transported to cytoplasmic fibers of the NPC that mediates bidirectional transportation. Then RanGTP was hydrolyzed to RanGDP by RanGAP1 resulting in the presence of RNA in the cytoplasm (Lin et al., 2015). Salt stress induced the up-regulation of RanGAP1 in *P. tomentosa*, suggesting that RanGAP1 might be salt responsive genes. In addition, the qRT-PCR result indicated its higher expression after salt treatment. Mutation of *RNA1*, which encodes RanGAP1, was reported to cause the accumulation of tRNA (Hopper and Banks, 1978) and rRNA in the nucleus (Hurt et al., 1999), which cause the damned hurt of living cell. It is therefore conclude that the up-regulated RanGAP1 may increase the efficiency of RanGTP hydrolysis and decrease the toxic effects of RNA accumulation. RanGAP1 plays an essential role in exchanges of genetics information, which might have a close relationship with following translation.

Ribosomes are the fundamental units that translate genetic information into proteins. NMD3, N-terminus with C_x2_C repeats and anuclear localization sequence, and C-terminus with a nuclear export sequence, is vital for ribosome assembly and for maintaining the efficiency of normal protein synthesis (Shi et al., 2014). In our study, NMD3 was enriched into ko03013, where its roles played in transport factor of 60S biogenesis and an adaptor for 60S subunit export, as well as a receptor in the cytoplasm were detected. Additionally, NMD3 contains nuclear shuttling sequences (Bühlmann et al., 2015). The detection of NMD3 indicates the conservation of this protein as a pre-60S nuclear export adaptor. An imperative mechanism of 60S ribosome maturation is the detachment of NMD3 from pre-60S particles, which triggers their combination with 40S subunits, the formation of rRNA precursors, and the constitution of fully functional ribosomes. A dominant negative form of NMD3, which had a truncated nuclear localization sequence, decreased the efficiency of protein synthesis as reflected by reduced cellulose content and variable sugar composition (Chen et al., 2012). The ratio of NMD3 proteins to free 60S subunits regulates 60S export to the cytoplasm and activates the expression of specific proteins in downstream operations (Lo and Johnson, 2009). In our study, *NMD3* was only up-regulated in PT4S. Additionally, the qRT-PCR result of NMD3 under salt stress indicated the higher expression, which might promote the “RNA transport” pathway to expedite the synthesis and expression of salt responsive proteins. From the annotation results of DEUs, bZIP TF families, ZIPs, ABCs, and DRR206 which might involved in osmosis and intracellular ion homeostasis were identified in *P. tomentosa* under salt environment.

### BZIP Tf families regulates ABA under salt stress

The DNA-binding domain of the bZIP TF families has a highly conserved C-terminal sequence and N terminus that is related to trans-activation activity. The DEUs coding bZIP TFs were up-regulated (Table S2) in the salt-treated *P. tomentosa* plants. Several bZIP gene TF families members, *bZIP14 TFs, bZIP26 TFs, bZIP37 TFs, bZIP50 TFs*, and *bZIP57 TFs*, were identified from PT4S vs. PT4 which enriched into the pathway of “Plant hormone signal transduction” (ko04075). While only *bZIP37 TFs* was differently expressed in the PT2S vs. PT2. In *Arabidopsis*, the bZIP14 and bZIP37 TFs were classified into Group A and the bZIP26, bZIP50, and bZIP57 TFs locating in C-terminal were classified into Group D(Jakoby et al., 2002). Group A were involved in ABA or stress signal response through the *cis* elements (Lopez-Molina and Chua, 2001). In transgenic *Arabidopsis*, the over-expression of *ZmbZIP72* contributed to increase salt tolerance by the positive modulation of an ABA-dependent TF (Ying et al., 2012), and this was reflected in some physiological indexes, such as leaf water loss, electrolyte leakage, proline content, and survival rates that were consistent with our results. Additionally, the activation of bZIP TF families is vital to ABA-dependent phosphorylation (Yoshida et al., 2009), which induces various tolerance responses. Almost all the DEUs coding for bZIP TFs were strongly up-regulated, and the qRT-PCR results of bZIP57 TFs and bZIP37 TFs also have high expression in the salt-stressed plants, which might effectively activate downstream ABA-inducible gene expression by binding to a major *cis*-acting element. The TFs in Group D were reported to participate in defense against pathogens by their interaction with TGA TFs and integrating different systemic signals (salicylic acid and ethylene) (Jakoby et al., 2002).

### Compatible ion transporter related to intracellular iron homeostasis

DEUs coding zinc transporters (ZIPs) were up-regulated in PT4S vs. PT4 but down-regulated in PT2S vs. PT2 (Table S4). We identified three unigenes, *ZIP1, ZIP4*, and *ZnT5* in the transcriptome libraries. ZIP1 is a member of the solute carrier family 39 that contains the main zinc transporters. ZIP4 associating with “Mineral absorption” (ko04978) is a bidirectional zinc transporter that locates in the endoplasmic reticulum. The function information of ZnT5 is not clear (Schneider et al., 2015). Zinc is a transition metal required by all living organisms. It is an important cofactor of nearly 300 enzymes in many metabolic pathways and is essential for maintaining the functional and structural integrity of cells (Wang et al., 2010). In *Arabidopsis*, A *ZIP1* mutant showed salt hypersensitivity in plant growth because zinc entry into the cytoplasm was inhibited (Cao et al., 2001). Intracellular zinc depletion induces cell apoptosis, and this can be mediated by the increased expression of ZIP genes under salt stress (Cao et al., 2001). The expression level of ZIP genes varied in different ploidy *P. tomentosa*. *ZIP1* was up-regulated in PT4S, suggesting that the adverse effects of salt stress may be mitigated by maintaining the zinc nutritional status(AbdEl-Hady, 2007), decreasing the uptake of salt ions (Cakmak and Marschner, 1988), and avoiding damage from free radical (Cakmak, 2000), which would preserve zinc homeostasis and help catalyst system achieving tolerant growth.

Transport proteins can transport stress-inducing ions from plant cells back into the rhizosphere, and sequester the stress-inducing ions into vacuoles (Jayakannan et al., 2015). Many DEUs coding members of the ABC transport family annotating into ABC transporters (ko02010) were identified in *P. tomentosa*, indicating that they may act as broad-spectrum salt resistance substances. ABC proteins are channel regulators that can regulate other channels, including an outwardly rectifying Cl^−^ channel, a Ca^2+^ activated chloride channel, a Na^+^ channel, and an inwardly rectifying K^+^ channel (Theodoulou, 2000). When many Na^+^ and H^+^ ions enter plant cells, they cause a net depolarization of the plasma membrane, which results in the bulk of NaCl-induced K^+^ loss under salt stress (Jayakannan et al., 2015). Additionally, the qRT-PCR results of ABCG15, ABCG31 ZIP1, and ZIP4 showed the high expression after salt stress. Therefore, transport proteins can play important roles in maintaining ionic equilibrium when plants are subjected to salt stress.

### Up-regulated DRR206 involved in plants disease resistance

The association of salt stress with disease resistance in plants is of interest. In our study, we identified *DRR206* annotating into “plant-pathogen interaction” pathway (ko04626) were up-regulated after salt treatment (see Table S4). DRR206 can activate hypersensitive responses including phytoalexin and other pathogenesis-related protein synthesis, as well as antioxidant redox systems, which can protect plant growth from pathogen damage under salt stress (De Gara et al., 2003). The promoter of the pea *DRR206* gene was reported to regulate elicitor-coding genes, like the β-glucuronidase reporter gene and the DNase elicitor gene, to confer disease resistance (Choi et al., 2004). DRR206 through its N-glycosylation activity can effectively induce the synthesis of the isoflavonoid that plays a positive role in the plant-pathogen signaling pathway (Melo-Braga et al., 2012; Celoy and VanEtten, 2014; Seneviratne et al., 2014). This activity may contribute to the growth of *P. tomentosa* under salt stress.

## Author contributions

RNA sequencing analysis and initial discovery of the DEUs did by GF and LW. XZ is responsible for material preparation. LW did RNA preparations and qPCR. GF, LW, MD, YD, ZZ, and YL took charge writing and modifying the research article. The final version of the manuscript was reviewed by all authors. All authors read and approved the final manuscript.

### Conflict of interest statement

The authors declare that the research was conducted in the absence of any commercial or financial relationships that could be construed as a potential conflict of interest.
