# Supplementary material for: Changes in Transcript Related to Osmosis and Intracellular Ion Homeostasis in Paulownia tomentosa under Salt Stress
Source: Front Plant Sci. 2016 Mar 30;7:384. doi: 10.3389/fpls.2016.00384 (PMC4813090; doi:10.3389/fpls.2016.00384)
Supplement: Supplementary file 1 [file DataSheet1.DOCX]

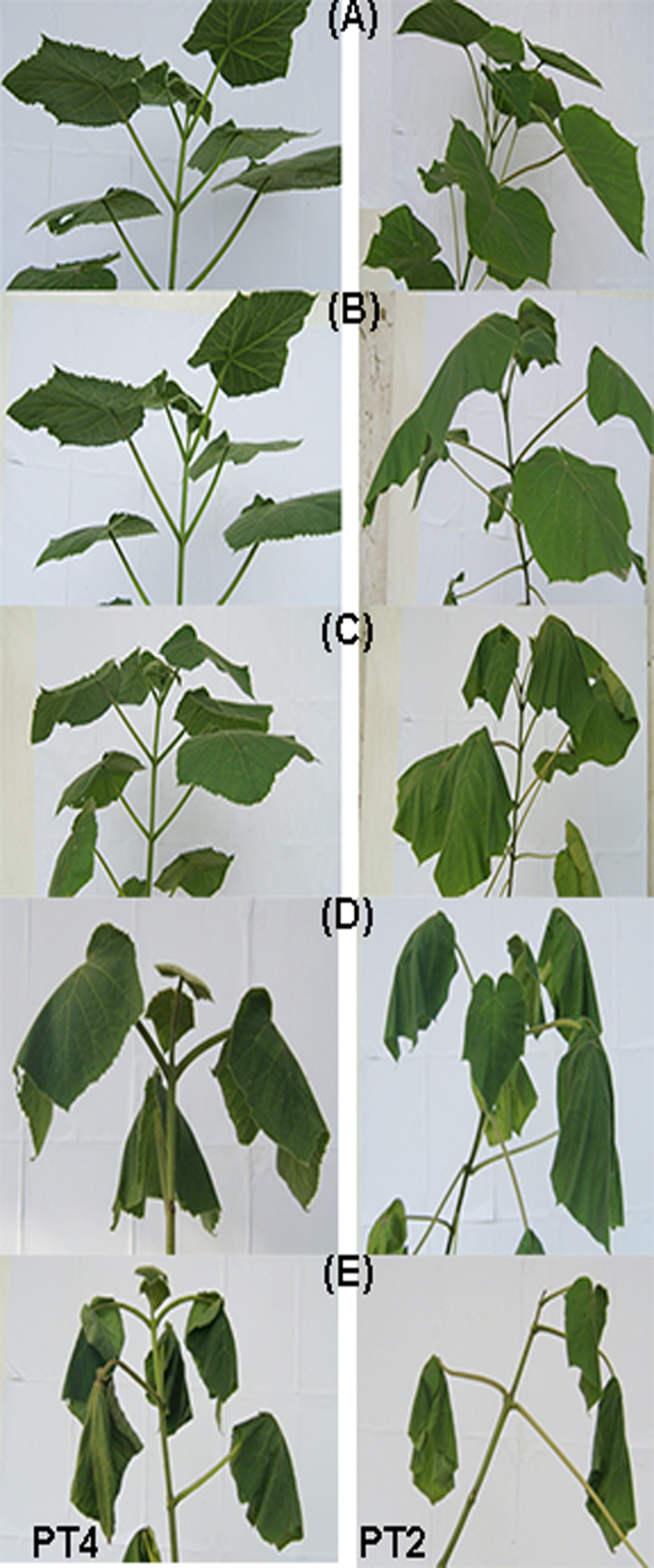


**Figure S1 Morphological features of diploids and tetraploids in response to salt stress**

PT2 represents diploid *Paulownia tomentosa*, PT4 represents autotetraploid *Paulownia tomentosa*. The right plant is PT2, and the left plant is PT4. (**A**) PT4 and PT2 were planted in the control soil; (**B**) PT4 and PT2 were treated with 70mM NaCl for 5d; (**C**) PT4 and PT2 were treated with 70mM NaCl for 10d; (**D**) PT4 and PT2 were treated with 70mM NaCl for 15d; (**E**) PT4 and PT2 were treated with 70mM NaCl for 20d.

**
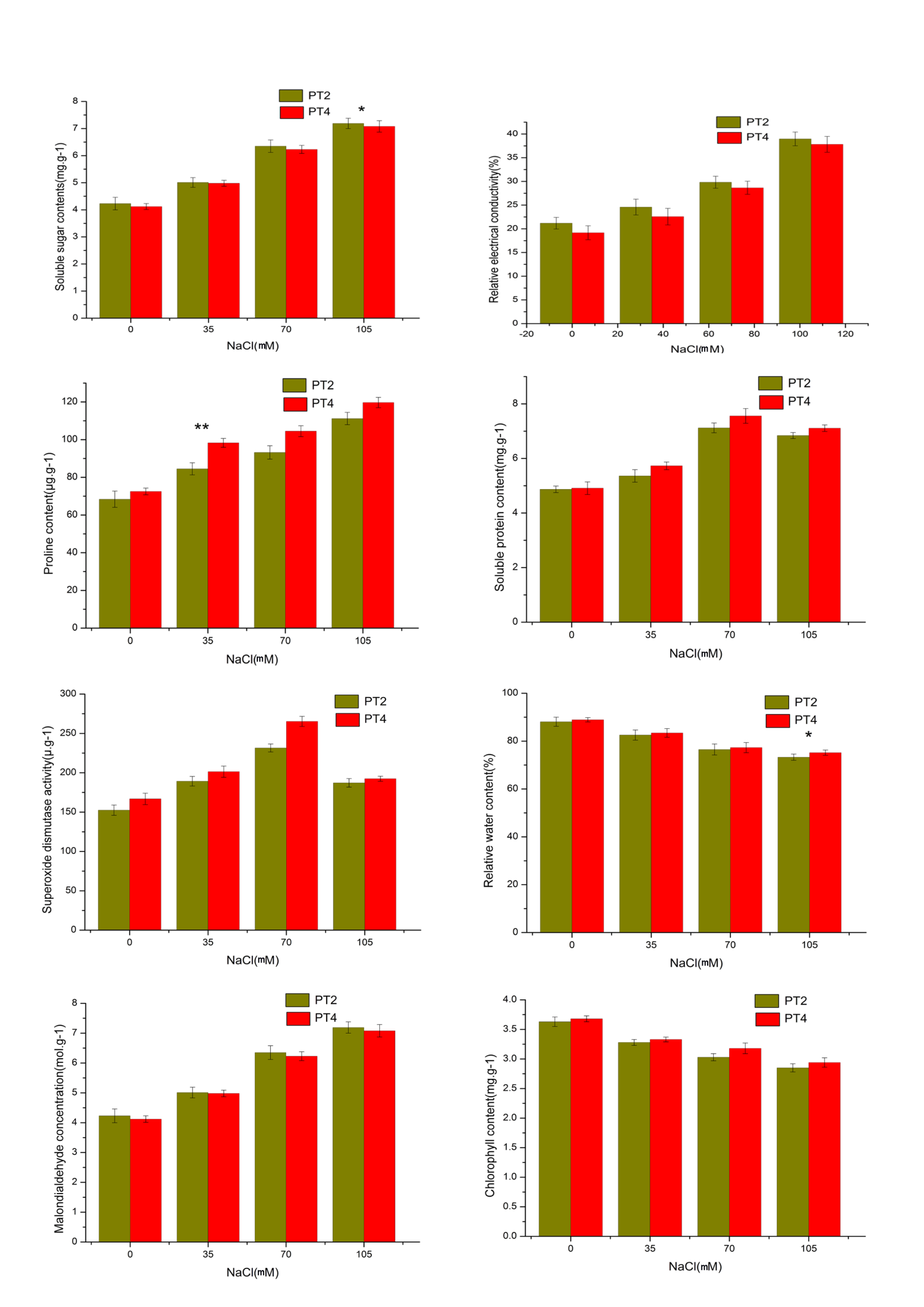
**

**Figure S2 Effects of salt stress on *P. tomentosa* physiology.**

PT2 represents diploid *Paulownia tomentosa*, PT4 represents autotetraploid *Paulownia tomentosa*.0, 35, 70, and 105mM NaCl were treated for 15 days. *,P<0.05.**,P<0.01. The significant analysis was used paired samples Test by SPSS 19.0.


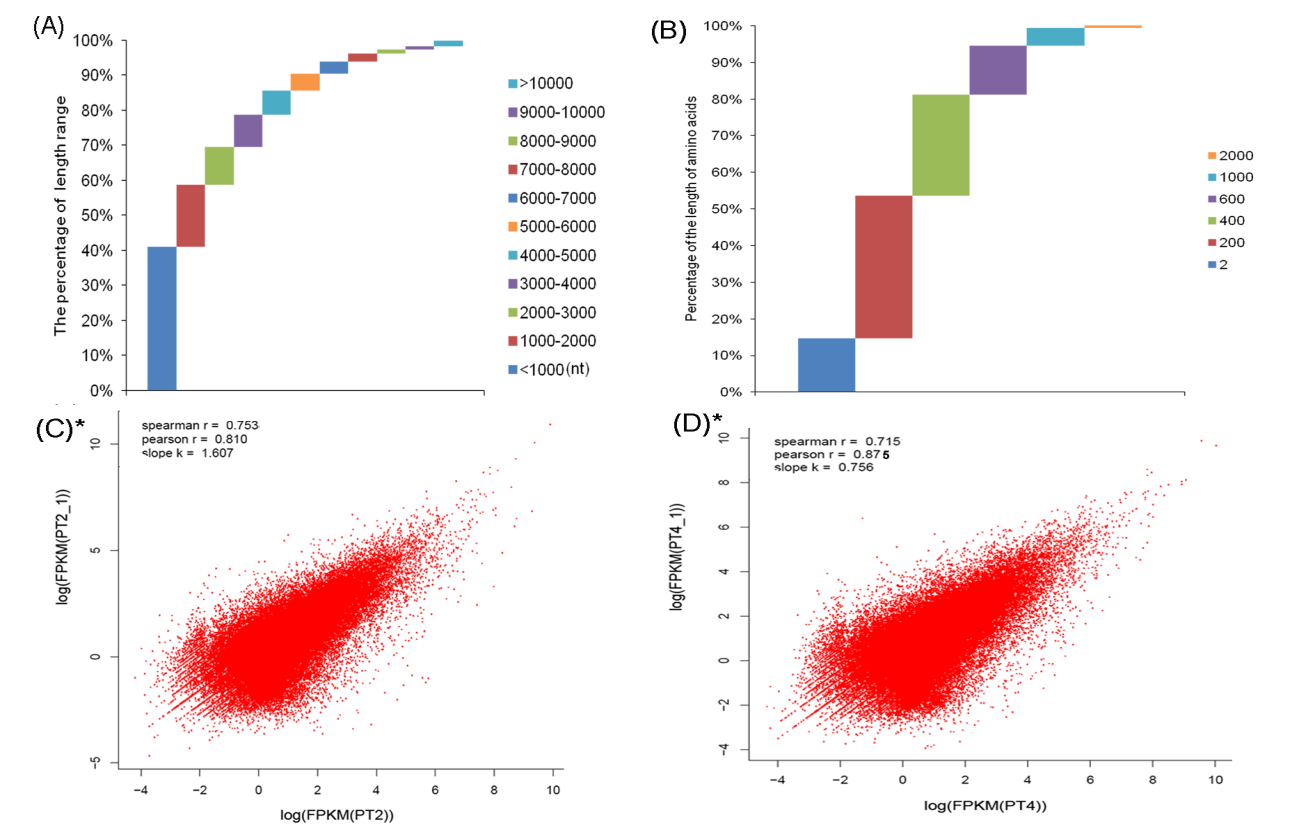


**Figure S3 The distribution of sequencing results.** A: The length distribution of the unigenes; B:The length distribution of CDSs’ amino acids; C: correlation coefficients of the expression of duplicate diploid *p. tomentosa*; D: correlation coefficients of the expression of duplicate autotetraploid *p. tomentosa*. X-axis represents the logarithmic value of diploid(C) or autotetraploid *p. tomentosa* (D) expression, while Y-axis represents the logarithmic value of the corresponding duplicate samples. * represents referenced figures from our previous article( Dong, Y., Fan, G., Deng, M., Xu, E., and Zhao, Z. (2014a). Genome-wide expression profiling of the transcriptomes of four Paulownia tomentosa accessions in response to drought ☆. Genomics. 104, 295-305).


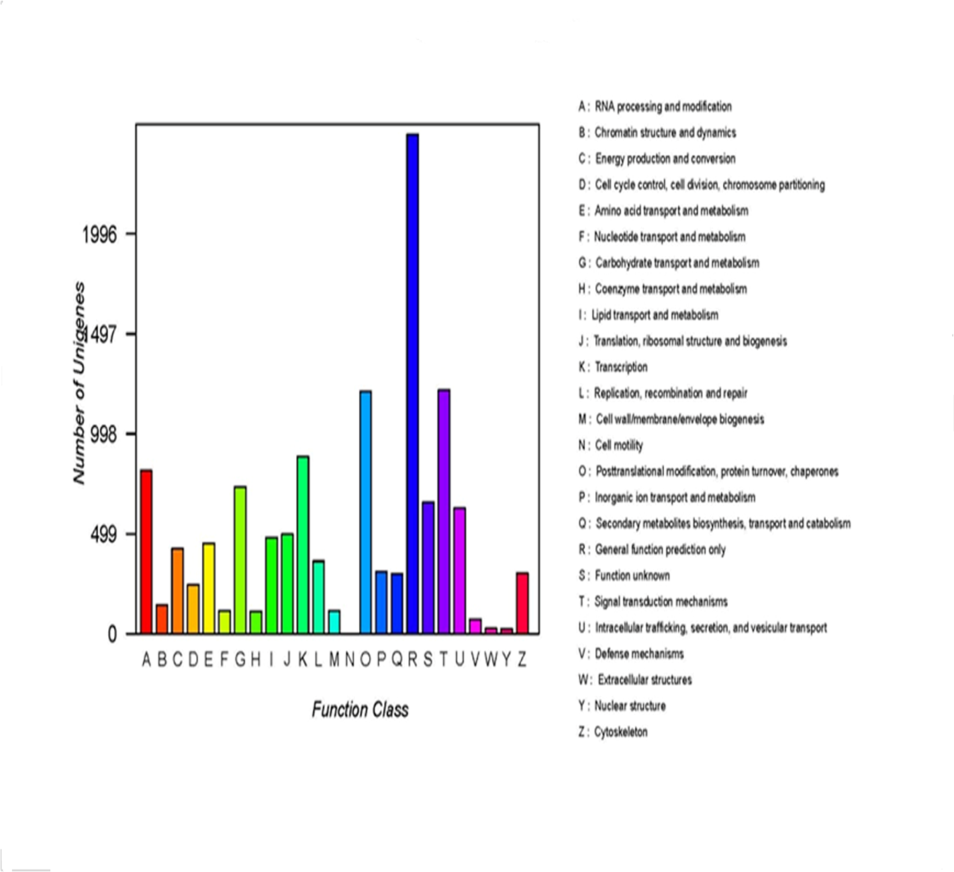


**Figure S4 The KOG function classification of all-unigenes**


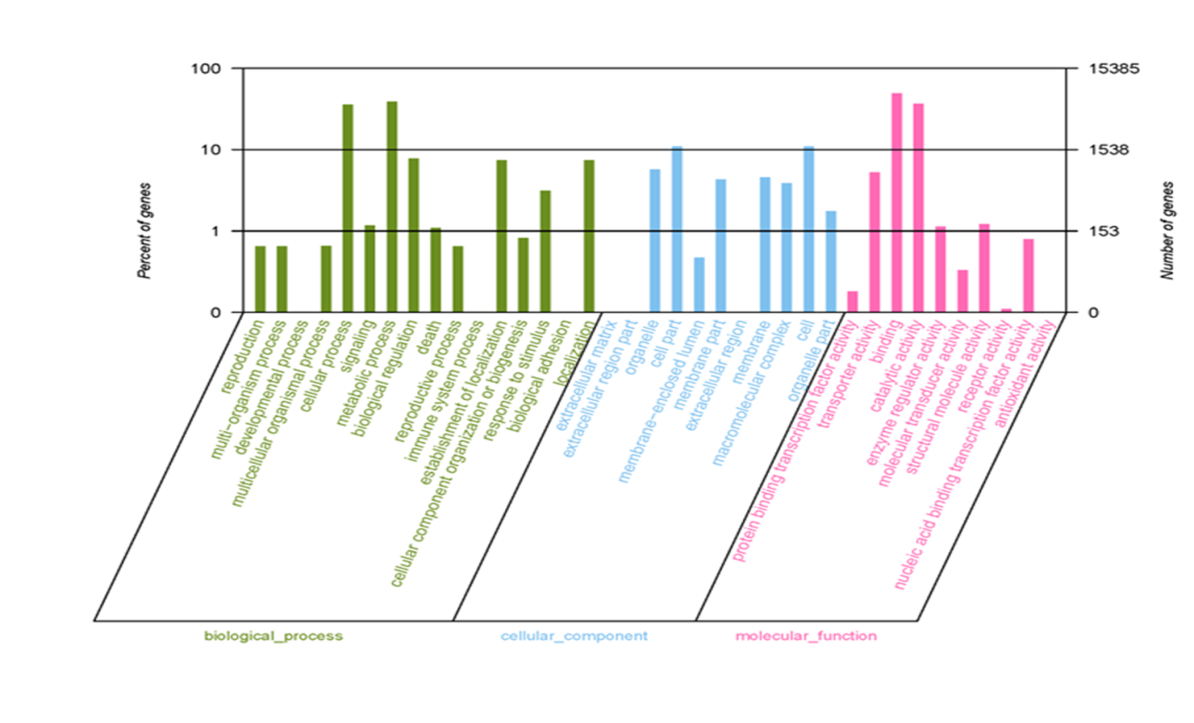


**Figure S5 The functional categories in the GO database under the three main functional categories (****biological process, cellular component, molecular function)**


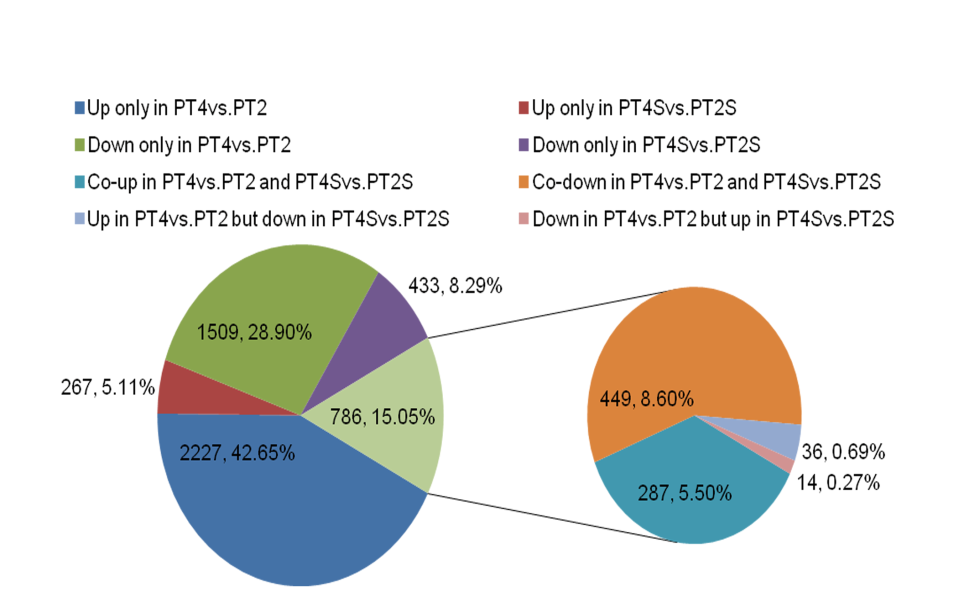


**Figure S6 The consistently differential expression unigenes in the PT4*vs*.PT2 and PT4S*vs*.PT2S comparisons**

786 DEUs were clustered into eight categories according to their expression patterns.

**
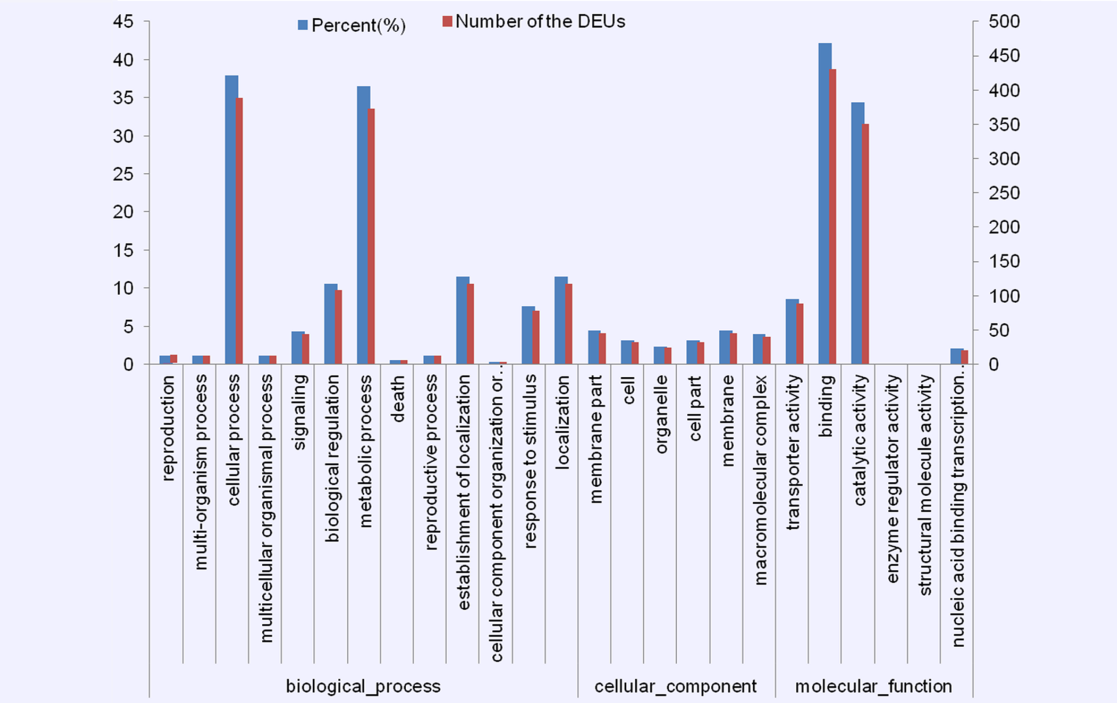
**

**Figure S7 The GO enrichment of the differential expression unigenes in the comparisons of PT2S*vs.*PT2 and PT4S*vs.*PT4.**

**
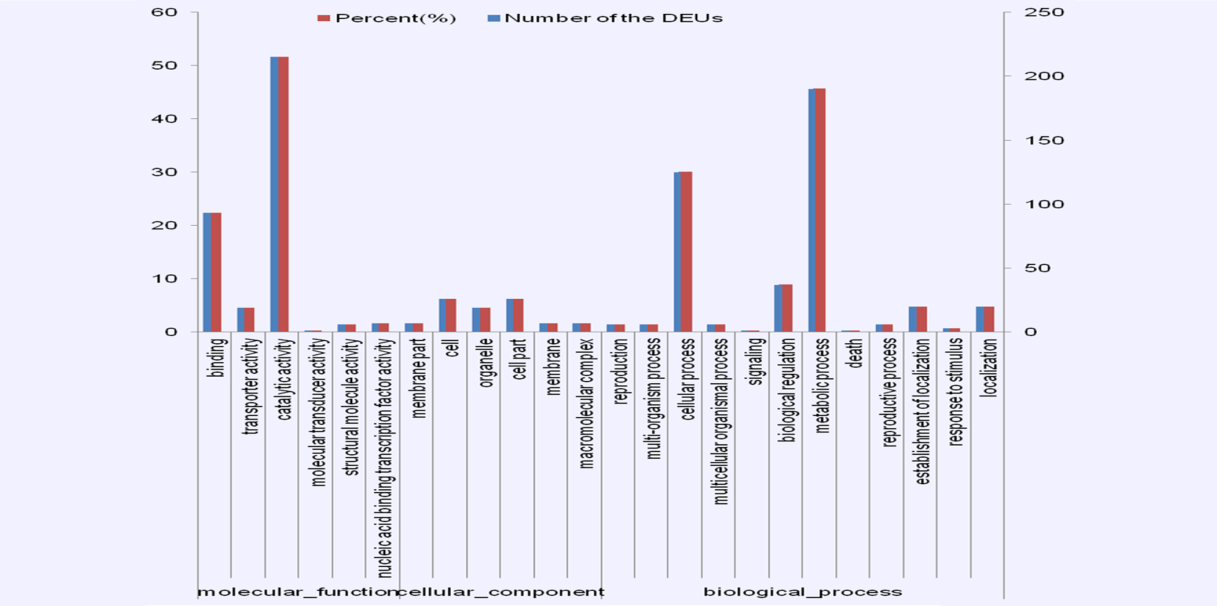
**

**Figure S8 The GO enrichment of the differential expression unigenes in the comparisons of PT4*vs.*PT2 and PT4S*vs.*PT2S**
